# The Effects of Oncolytic Pseudorabies Virus Vaccine Strain Inhibited the Growth of Colorectal Cancer HCT-8 Cells In Vitro and In Vivo

**DOI:** 10.3390/ani12182416

**Published:** 2022-09-14

**Authors:** Chunxia Chai, Jinlong Zhang, Yanyan Zhou, Hua Yin, Fan Zhang, Yun Diao, Xiaohui Zan, Yanhua Ma, Yan Wang, Youzhi Wu, Wei Wang

**Affiliations:** 1 State Key Laboratory of Reproductive Regulation and Breeding of Grassland Livestock, School of Life Sciences, Inner Mongolia University, Hohhot 010000, China; 2 Inner Mongolia Academy of Agricultural and Animal Husbandry Sciences, Hohhot 010000, China; 3 Basic Medical School, Inner Mongolia Medical University, Hohhot 010000, China

**Keywords:** pseudorabies virus, colorectal cancer, oncolytic, transplant model, apoptosis

## Abstract

**Simple Summary:**

Oncolytic viruses have emerged as a viable therapy for cancers, and we explored the oncolytic effects of pseudorabies virus on colorectal cancer cells. The replication capacity and cytotoxic effect of the virus were investigated on colorectal cancer cells, meanwhile, the antitumor ability and safety were evaluated in a mouse tumor transplantation model tumor transplantation. For the first time, we found that the attenuated live vaccine strain of pseudorabies virus used in this study showed better anti-tumor activity. These results set the stage for subsequent clinical trials of tumor therapy using oncolytic viruses in non-primates and humans.

**Abstract:**

Oncolytic viral therapy is a promising treatment approach for a variety of tumor forms. Although a number of studies have demonstrated that the pseudorabies virus (PRV) may be applied as an oncolytic carrier, the anti-colorectal cancer impact of the virus and the mechanism of its cytotoxic effect remain elusive. In this study, the replication capacity and cell activity of PRV attenuated live vaccines Bartha K61 and HB98 in HCT-8 cells in vitro were investigated. Next, the antitumor ability and safety were evaluated in a mouse model of HCT-8 tumor transplantation. Both PRV strains were able to suppress tumor growth and HB98 showed higher safety and efficiency than the Bartha K61 strain. Finally, flow cytometry and immunohistochemistry examination were performed to investigate its possible cytotoxic mechanism. The results showed that PRV inhibited tumor proliferation both in vitro and in vivo by inducing apoptosis. In summary, our study discovered for the first time that the live attenuated PRV has an oncolytic effect on HCT-8 cells with high efficacy and safety.

## 1. Introduction

According to the most recent global cancer statistics report, cancer is quickly increasing in incidence and death globally, and it will soon become the leading cause of death, posing a severe threat to people’s ability to longer life-span of human [1]. Colorectal cancer (CRC) has an incidence of 10.0% and a fatality rate of 9.2% in global data, placing it third and second in terms of cancer morbidity and mortality, respectively [1]. Despite great advances in detection techniques, around one-fifth of CRC patients have tumor metastasis at the time of diagnosis, leading in a considerable reduction in overall survival (OS). Oncolytic viruses (OVs) have emerged as a viable therapy for cancers. At present, some OVs including the vaccinia virus, herpes simplex virus, vesicular stomatitis virus, measles virus, tanapox virus, echovirus, reovirus, and Newcastle disease virus have been tested in against CRC [2].

OVs therapy is a potential therapeutic strategy for cancers. Natural or genetically engineered viruses can directly or indirectly infect, transduce, and thus kill cancer cells through the transmission of microenvironment cells. In addition to directly killing cancer cells, OVs can also regulate the host immune system to enhance cancer suppression and eradication [3]. The dominant characteristic of OVs is that their selective replication in tumor cells is higher than in normal cells, especially for those of transgenic strains [4]. A common strategy is to delete certain virulence-determined genes, such as the thymidine kinase (TK) gene of pseudorabies virus (PRV). TK is a necessary gene for viral DNA replication. The deletion of the TK gene may make some viruses more selective to cancer cells. In some OVs, the deletion of this gene leads to an increase in tumor selectivity and solves the concern of uncontrolled replication in non-target tissues [5,6,7].

PRV, an important viral pathogen of pigs, belongs to the *Herpesviridae* family and *Alphaherpesvirinae* subfamily. It is an important viral pathogen of pigs. It can cause nervous system diseases in young piglets, respiratory diseases and growth retardation in fattening pigs, and reproductive disorders in sows [8]. Although PRV has a wide range of hosts, pigs (including other wild boars) are the only natural hosts [9]. In recent years, it has been reported that PRV mutants can infect humans and cause encephalitis and endophthalmitis, but no human-to-human transmission has been found [10,11,12]. These cases seem to be related to highly pathogenic PRV emerging variants in China, where PRV vaccination is not mandatory among swineherds. PRV encoded glycoproteins D (gD), E (gE) and I (gI) are important for the interaction between virus and host cells. gE protein is indispensable for viral replication but is necessary for the cross-neuronal transmission of PRV. Deletion of gE reduces virulence because of the reduced ability to infect secondary and tertiary neurons in the olfactory and trigeminal pathways in the nervous system [13]. The nonstructural protein TK supports viral DNA replication and plays an important role in virulence and neuroinvasiveness. PRV Bartha K61 strain is one of the first attenuated vaccines ever developed, it contains a large deletion in the US portion of the genome, resulting in full deletion of the genes encoding gE and US9 and a partial deletion of the genes encoding gI and US2. Interestingly, it was later revealed that there is a strong selection toward gE deletion [14]. PRV HB98 strain, a triple gene deletion strain of TK^−^/gG^−^/gE^−^, is an approved vaccine strain with strong immunogenicity, which has been widely used to prevent and control PRV infection in China [15]. In addition, it is necessary to produce infectious virions in non-mitotic cells [16]. Other members of the *Alphaherpesvirus* subfamily have been studied for their potential oncolytic properties, such as HSV-1, HSV-2, and BHV-1 [17,18]. Recently, the first oncolytic HSV-1, talimogene laherparepvec (T-VEC, also known as OncoVEXGM-CSF) has been approved in the United States, Europe, and Australia for the treatment of melanoma patients with advanced melanoma that has recurred after initial surgery [19,20,21].

In this study, we determined the ability of PRV to replicate, cause cytopathic effect, and affect cell viability in human colorectal cancer cell line HCT-8. In the tumor model of HCT-8 xenotransplantation in mice, we found that PRV could significantly inhibit tumor growth, and its safety varied according to the deletion of viral virulence determine genes. This is the first time a report has proved that PRV can work as a novel oncolytic virus with the potential for the treatment of colorectal cancer cells.

## 2. Materials and Methods

### 2.1. Cells and Virus

Human colon cancer cell line HCT-8 cells were purchased from China Center for Type Culture Collection and cultured in RPMI 1640 medium (Gibco, Carlsbad, CA, USA) containing 10% FBS (Gibco, Carlsbad, CA, USA) and 100 U/mL penicillin-streptomycin. VERO (African green monkey kidney) cells were purchased from the China Center for Type Culture Collection and cultured in DMEM medium (Gibco, Carlsbad, CA, USA) containing 10% FBS. PRV Bartha K61 strain and PRV HB98 strain are widely used live attenuated vaccine strains, which were purchased from China Animal Husbandry Industry Co., Ltd., (Beijing, China) and Wuhan Keqian Animal Biological Products Co., Ltd., (Wuhan, China) respectively. The PRV Bartha K61 strain lacks primarily the gE and gI genes, whereas the PRV HB98 strain lacks the TK, gG, and gE genes [22,23]. The viruses were propagated in VERO cells and stored at −70 °C until use.

### 2.2. In Vitro Virus Replication and Virus Titration

PRV was inoculated with a multiplicity of infection (MOI) of 1 and incubated in HCT-8 cells at 37 °C for 2 h in a 5% CO_2_ incubator. The supernatant was discarded and the fresh DMEM medium was added. The virus was collected at 24 h, 48 h, and 72 h, respectively. The tissue culture infectious dose (TCID_50_) was determined by 50% endpoint dilution determination according to the Reed and Muench method, and the titer of the virus was calculated in VERO cells [8].

### 2.3. Evaluation of Cytopathic Effects (CPEs) In Vitro

PRV was inoculated into HCT-8 cells at an MOI of 1 and incubated at 37 °C for 2 h. The virus solution was discarded and the fresh DMEM medium was supplemented. The CPEs were observed at 48 h by an optical microscope (Nikon, Tokyo, Japan).

### 2.4. Cell Viability Assay

An amount of 2 × 10^5^ HCT-8 cells were inoculated on a 96-well culture plate (Corning, Corning, NY, USA) and cultured for 24 h, then incubated with PRV in RPMI 1640 medium with a certain MOI at 37 °C for 2 h. At the end of the incubation period, the virus-containing medium was removed, the cells were washed with buffered phosphate-buffered saline (Gibco, Carlsbad, CA, USA), and then a fresh medium was added. An amount of 10 µL CCK-8 (Proteintech, Chicago, IL, USA) solution was added to each well, and the culture plate was incubated in the incubator for 1~4 h. The absorbance at 450 nm was measured by a microplate reader (BioTEK, Winooski, VT, USA).

### 2.5. Animal Experiments

Five-week-old female BALB/c nu mice and BALB/c mice were purchased from SPF Beijing, Biotechnology Co., Ltd. (Beijing, China). All mice were placed in standard conditions and had free access to food and water. After 6 weeks, 1 × 10^7^ HCT-8 cells were injected subcutaneously under the right flank in BALB/c nu mice. When the tumor was palpable (50~100 mm^3^), the mice were randomly divided into 3 groups with similar tumor volumes. The mice received 100 µL of PRV Bartha K61 (1 × 10^7^ TCID_50_/_mL_), PRV HB98 (1 × 10^7^ TCID_50_/_mL_), and PBS in the control group (100 µL) twice a week for a total of two weeks, respectively. Every other day, the tumor size was measured with Vernier calipers. Tumor volume was calculated as V = 0.5 × (length × width^2^), and body weight was recorded. Mice were euthanized if they appeared moribund or the tumors exceeded 20 mm in maximum diameter. After 22 days of treatment, the surviving mice were euthanized, and tumor tissues were fixed with 4% paraformaldehyde and embedded in paraffin. Tumor sections were stained with hematoxylin–eosin (HE) and immunohistochemistry (IHC).

BALB/c nu mice and BALB/c mice were injected intravenously (i.v.) with PRV Bartha K61 or PRV HB98 strain at 1 × 10^8^ TCID_50_, PBS was used as control. The body weight and survival were evaluated for PRV’s safety.

PRV was injected intratumorally at 1 × 10^7^ TCID_50_ in BALB/c nu mice bearing HCT-8 tumors, PBS was used as control. Various tissues including blood, brain, lung, liver, heart, kidney, and tumor were collected 12 h and 120 h after injection for qPCR detection.

### 2.6. Ethics Statement

All animal experiments were carried out in strict accordance with the recommendations in the “Guidelines for the Ethics and Use of Laboratory Animals” by the Ministry of Science and Technology of the People’s Republic of China. The protocol in this study was approved by the Institutional Animal Ethics and Use Committee of Inner Mongolia University (IMU-MOUSE-2021-033, 26 July 2021).

### 2.7. Real-Time PCR for the Detection of PRV 

DNA were extracted using the QIAamp DNA Mini Kit (QIAGEN, Dusseldorf, Germany). The products were amplified on LightCycler^®^ 480 instrument (Roche Applied Science, Basel, Switerland) with TB Green ^®^Premix Ex Taq II (TAKARA, Ōsaka Japan), according to the manufacturer’s instructions. The primers used were as follows: forward, 5′-CGGTCACCTTGTGGTTGTTGC-3′ and reverse, 5′-AGGGGATCGCCGTGCTCT-3′. The reaction protocol was 30 s at 95 °C, 40 cycles of 5 s at 95 °C and 30 s at 60 °C. The results were presented as the mean ± SD from three experiments.

### 2.8. Histological Examination

For light-optical examination, tumor tissue samples were fixed in 10% formalin at 4 °C for three days. According to a standard protocol, the samples were dehydrated in a series of graded concentrations of alcohol, butanol, and xylene, and embedded in paraffin. The obtained paraffin blocks were divided into 4.5~5 µm thick sections. Deparaffinized sections were subjected to hematoxylin–eosin overview staining according to the standard procedure and embedded in a Vitrogel mounting medium field [24]. The stained samples were examined on an upright optical microscope (Nikon, Japan).

### 2.9. Immunohistochemistry

Tissue sections were deparaffinized with xylene and rehydrated through a series of graded ethanol. For antigen unmasking, sections were immersed in 10 mM sodium citrate buffer (pH 6.0) and heated for 10 min in a microwave. Endogenous peroxidase was quenched in 1% hydrogen peroxide for 10 min. The sections were blocked by incubation with 5% normal goat serum for 1 h at room temperature [24]. Anti-cleaved Caspase-3 rabbit polyclonal antibodies (Servicebio, Wuhan, China) were incubated overnight at 4 °C. Immunostaining with HRP-conjugated secondary antibodies was continued (BioWorld, MN, USA). Incubation was performed for 10 min at room temperature according to the manufacturer’s instructions. DAB Chromogen (Servicebio, Wuhan, China) was used for incubation at room temperature for 4 min after washing. Each organ section was stained with hematoxylin for 2 min, dehydrated with ethanol and xylene, patched, and slide covered. The nucleus of hematoxylin stained is blue, and the positive expression of DAB is brownish yellow. The integrated optical density (IOD) of the IHC section was calculated by Image Pro Plus 6.0 (Media Cybernetics, MD, USA).

### 2.10. Flow Cytometry

The ratios of apoptotic cells were further determined by flow cytometric analysis using an Annexin V-FITC apoptosis detection kit (BD Biosciences, Franklin Lakes, New Jersey, USA) according to the manufacturer’s instructions. HCT-8 cells were seeded in a 6-well plate at a density of 1 × 10^6^ cells/well and exposed to PRV (MOI = 1) or 1640 medium for 24 h, 48 h, 72 h, and 96 h. Apoptosis of the stained cells was detected using a LSRFortessa flow cytometer (BD, FACSCalibur, Franklin Lakes, New Jersey, USA). Flow Jo Software (Tristar, California, USA) was used for analysis.

### 2.11. Statistical Analysis

The data were presented as means, with error bars representing the mean’s standard deviation (SD). Differences between groups were compared using one way ANOVA and two-tail Student’s test. Survival was calculated by Kaplan–Meier method, and differences between curves were assessed by log-rank test. *p* values less than 0.05 are significant. GraphPad Prism 8™ (GraphPad Software, USA) was used for all the statistical analyses.

## 3. Results

### 3.1. Replication of PRV in HCT-8 Cells

**Figure 1:**
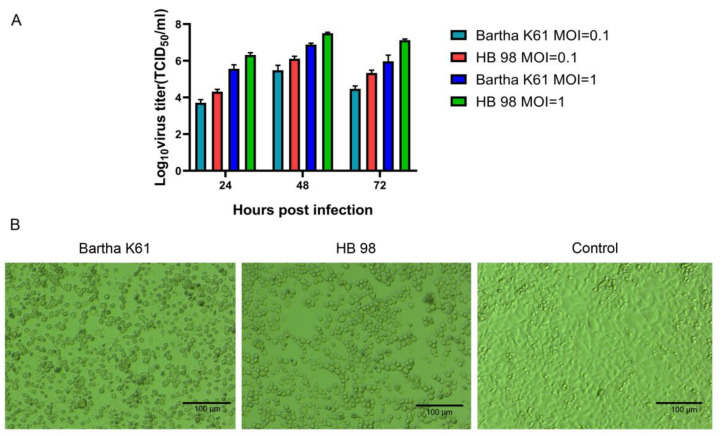
PRV infected HCT-8 cells. (**A**) PRV in the cell culture supernatant was collected separately at different time points, and the virus titer was determined by the TCID_50_ assay. Data represent mean ± SD from biological triplicates. (**B**) HCT-8 cells were infected with PRV Bartha K61 strain and HB 98 strain at an MOI of 1. The images were obtained at 48 h after infection.

To investigate whether PRV is capable of infecting and replicating in HCT-8, we used two PRV vaccine strains with different gene deletions. PRV Bartha K61 strain naturally lacks the gE and gI genes through viral passaging, while the PRV HB98 strain is devoid of TK, gG, and gE genes through genetic engineering. Both strains were inoculated with an MOI of 0.1 TCID_50_ and an MOI of 1 TCID_50_ into HCT-8 cells and incubated for 2 h to ensure the binding of the viruses. The supernatants of the two cultures were collected at 24 h, 48 h, and 72 h for the TCID_50_ assay, respectively (Figure 1A). Therefore, both PRV strains can infect and replicate in HCT-8 cells with high viral titers. 

To confirm that PRV induced CPEs, Both PRV strains were used to infect HCT-8 cells, and CPEs were observed at 48 h after infection (Figure 1B). The results showed that PRV had an oncolytic effect on HCT-8 cells.

### 3.2. In Vitro Cell Viability

**Figure 2:**
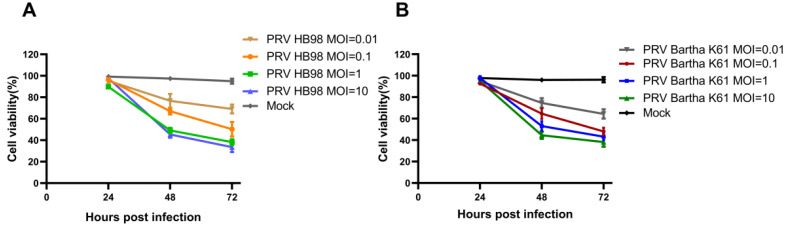
PRV infection on the viability of HCT-8 cells. (**A**) HCT-8 cells infected with PRV Bartha K61 strain (MOI = 0.01, MOI = 0.1, MOI = 1, MOI = 10) were cultured for different time points, and then cell viability was assessed by CCK-8 assay. (**B**) HCT-8 cells were infected with PRV HB98 strain at MOI of 0.01, 0.1,1, and 10 TCID_50_ and cultured for different time points, and then cell viability was assessed by CCK-8 assay. Data were presented as mean ± SD from three repeated experiments with five parallel repeats in each experiment.

To test the oncolytic potency of PRV, HCT-8 cells were infected with PRV (MOI = 0.01, MOI = 0.1, MOI = 1, MOI = 10), and the cell viability was analyzed by CCK-8 assay. As shown in Figure 2, PRV inhibited the proliferation of HCT-8 in a dose-and time-dependent manner. It was worth noting that the inhibitory effect was not obvious at 24 h post-infection (hpi). At an MOI of 1 and 10 TCID_50_, cells infected with PRV were less than 50% at 48 hpi, which indicated the high cytotoxicity of PRV to cells. The above results become more apparent at 48 hpi and 72 hpi. 

### 3.3. PRV Reduces Tumor Growth in BALB/c nu Mice Model

**Figure 3:**
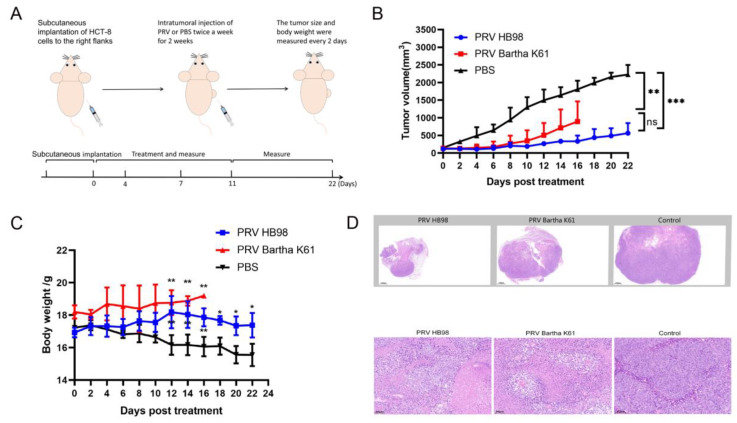
Oncolytic efficacy of PRV in BALB/c nu mice. (**A**) Treatment schedule. Six-week-old female BALB/c nu mice were injected with 1 × 10^7^ HCT-8 cells subcutaneously under the right flank. An amount of 100 µL of PBS or PRV (1 × 10^7^ TCID_50_/_mL_) was injected into the established tumor on days 0, 4, 7, and 11. The tumor size and body weight were measured every 2 days. (**B**) The mean tumor volume was shown, and the data were presented as mean ± SD (*n* = 6 /group). The statistical difference on day 22 between PRV-treated groups from PBS-treated groups was determined using *t* test. (** *p* < 0.01, *** *p* < 0.001) (**C**) The net body weight was measured, and the data were presented as mean ± SD (*n* = 6/group). * *p* < 0.05, ** *p* < 0.01. (**D**) Tumor sections were taken for HE staining in each group. The image displayed represents one mouse in each group, with 1.3× magnification and 20× magnification (scale bar = 50 µm). ns: not significant.

Next, we explored the anti-tumor effect of PRV in the BALB/c nu mice model of HCT-8 cells. The mice were randomly divided into three groups (*n* = 6) and treated with PRV Bartha K61 strain, PRV HB98 strain, and PBS twice a week for two weeks (Figure 3A). The tumor volume and net body weight were measured continuously after injection. On the 22nd day, the study was terminated since the average tumor size of mice in the PBS group reached 2000 mm^3^. After 5 days of initial treatment, the tumor size in the PBS group was significantly larger than that in the mice of the other two groups. Consistent with the in vitro results, PRV strain Bartha K61 and HB98 significantly inhibited in vivo tumor growth (Figure 3B). Similar to previous research [25], all mice of the PRV Bartha K61 group died within 16 days with itching and ulceration, while PRV HB98 treated mice survived longer, up to 22 days without any adverse reactions. When the tumors were harvested at the endpoint of the study (day 22), we found that the tumor size of mice in the treatment groups was significantly smaller than that in the PBS control group. These results suggested that PRV, as an oncolytic virus, had a significant anti-tumor effect. Compared with the PBS group, the average tumor volume in the PRV treatment groups decreased significantly. The body weight of the mice having increased tumor volumes in the control group decreased instead, while the body weight of the mice in PRV-treated groups was relatively stable, moreover, a significant difference has been found between the HB98 group and the PBS group (Figure 3C). These results indicated that PRV treatment can inhibit the rapid growth of tumors and the reduction of body weight in mice.

We evaluated the pathological difference of tumor cells in vivo between PRV treatment and the control group. Compared with the control group, the average necrotic area ratio of tumor cells in the PRV treatment group increased significantly (Figure 3D). In the control group, there was nest-like growth of tumor cells, nuclear atypia, giant nuclei, tumor-infiltrating lymphocytes, as well as more mitoses and necrosis. There were pyknosis, fragmentation, melting of the nucleus, and large area necrosis in the PRV HB98 group. In the Bartha K61 group, the tumor cells did not form large tumor nests. The tumor cells were swollen, deformed, ruptured, and necrosed.

### 3.4. Evaluation of the Safety of PRV In Vivo

**Figure 4:**
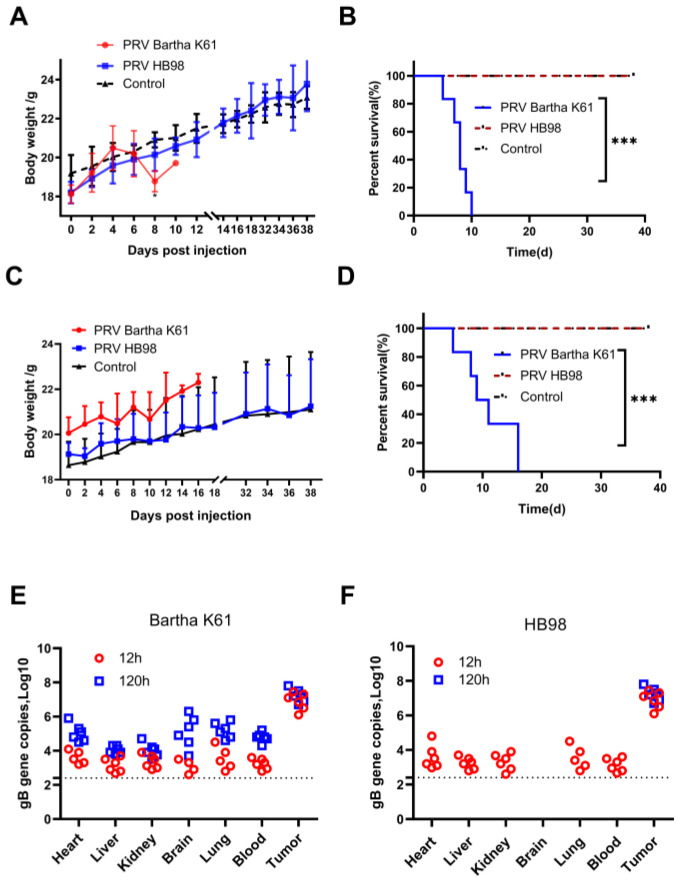
Safety and biodistribution of PRV in mice. (**A**) PRV Bartha K61 or HB98 strain was injected intravenously into BALB/c nu mice at 10^8^ TCID_50_, and PBS was used as a control. Body weight was monitored at the indicated time points; the data were presented as means ± SD (*n* = 6 mice/group). (**B**) Kaplan–Meier survival curves for mice treated with PBS and PRV (*n* = 6 mice/group). (**C**) PRV was injected intravenously into BALB/c mice at 10^8^ TCID_50_, and PBS was used as a control. Bodyweight was monitored at the indicated time points; the data were presented as mean ± SD (*n* = 6 mice/group). (**D**) Kaplan–Meier survival curves for immune-competent mice treated with PBS and PRV (n = 6 mice/group). *** *p* < 0.001. (**E**) PRV Bartha K61 strain of 10^7^ TCID_50_ was intratumorally injected into BALB/c nu mice bearing HCT-8 cells. The copy number of virus genome in different tissues were detected at 12 h and 120 h after injection. The dotted line represents the limit of detection (*n* = 6 mice/group). (**F**) HCT-8 cells were subcutaneously injected into BALB/c nu mice, and PRV HB98 strain of 10^7^ TCID_50_ was injected intratumorally. The copy number of viral genome in each tissue was detected at 12 h and 120 h after injection, and the dotted line represents the limit of detection (*n* = 6 mice/group).

Since the PRV Bartha K61 strain caused the death of mice, the safety of the two strains raised our concern. The safety of the two strains was further evaluated by measuring the body weight and survival of healthy non-tumor-bearing mice after administration of PRV HB98 and PRV Bartha K61, respectively. BALB/c nu mice and BALB/c mice were injected i.v. with PRV Bartha K61 and PRV HB98 strain at 1 × 10^8^ TCID_50_, PBS was used as control. The nude mice injected with PRV HB98 did not lose weight (Figure 4A) and survived healthily up to the 38th day (Figure 4B), in contrast, the mice injected with PRV Bartha K61 strain significantly lost weight and died all on the 10th day. On the other hand, in immune-competent BALB/c mice, there was no significant difference in body weight between the two PRV-treated groups (Figure 4C. However, the mice in the Bartha K61 group still died on the 16th day, while the mice in the HB98 group survived healthy (Figure 4D), which was consistent with the results in the immunodeficient BALB/c nu mice.

The biodistribution of PRV was assessed in tumor-bearing mice to determine the persistence and dissemination of tumors. PRV was administered intratumorally at 1 × 10^7^ TCID_50_ in BALB/c nu mice bearing HCT-8 tumors. Blood, brain, lung, liver, heart, kidney, and tumor tissues were collected at 12 and 120 h after injection. Genomic DNA (gDNA) was extracted and then quantified by qPCR targeting the PRV gB gene. In the Bartha K61 group, the copy number of the viral genome was detected in all tissues at 12 h and 120 h after injection, and the number of viral genetic material in the tumor was higher than that in other tissues (Figure 4E). In the HB98 group, virus genetic materials could be detected in all tissues except the brain at 12 hpi, but not at 120 hpi except in the tumor, which means that HB98 did not significantly spread from the tumor to other tissues. (Figure 4F). 

### 3.5. Pathological and Immunohistochemical Study of Colorectal Cancer Cells Treated with PRV

**Figure 5:**
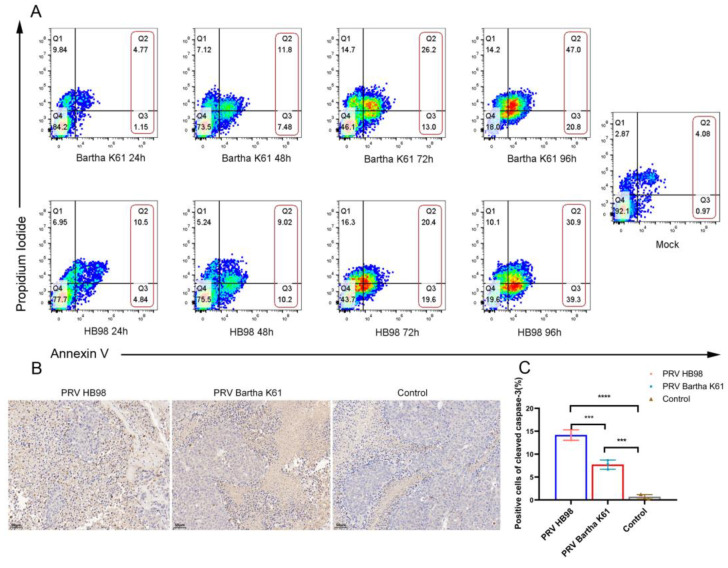
PRV induces apoptosis in HCT-8 cells. (**A**) HCT-8 were separately infected with PRV Bartha K61 or HB98 strain at an MOI of 1. Cells were stained with Annexin V/Propidium Iodide to determine the percentage of early and late apoptotic cells by flow cytometric analysis at different hours. Red boxes represent apoptotic cells. (**B**) Apoptosis of tumor cells was assessed and quantified by IHC (cleaved caspase-3), with 40× magnification (scale bar = 50 µm). (**C**) Positive cells ratio of cleaved caspase-3. The data were presented as mean ± SD (n =3/group). *** *p* < 0.001, **** *p* < 0.0001.

Finally, considering that HSV has an oncolytic effect on tumor cells by inducing apoptosis [26,27], the potential oncolytic mechanism of PRV was explored. Thus, apoptosis was examined by flow cytometry and IHC assay. After HCT-8 cells were infected with Bartha K61 strain or HB98 strain at an MOI of 1, the proportion of apoptotic cells was significantly increased in a time-dependent manner (Figure 5A). In HCT-8 cells, the apoptotic rate after PRV Bartha K61 infection was 6%, 19%, 39%, and 68% at 24 h, 48 h, 72 h, and 96 h, respectively; however, the apoptosis rate at the mock-treated group was 5.05%. In the HB98 group, the apoptotic rate after infection was 15%, 19%, 40%, and 70% at 24 h, 48 h, 72 h, and 96 h, respectively. IHC analysis of the tumor showed that the abundance of cleaved caspase-3 in the PRV-treated group was higher than that in the control group (Figure 5B). The positive cell ratio of cleaved caspase-3 was 14.17 ± 0.66% in the HB98 group, 7.71 ± 0.59% in the Bartha K61 group, and 0.70 ± 0.28% in the control group (Figure 5C), which suggested that the decrease in tumor volume is correlated with the apoptosis of tumor cells.

## 4. Discussion

OVs are naturally existing or genetically modified viruses, which can selectively infect cancer cells and cancer-related endothelial cells, where OVs can replicate and result in direct oncolysis. At present, there are many kinds of oncolytic virus strains are being evaluated in clinical trials, including MV, HSV, adenovirus, and poliovirus [28]. Studies have found that recombinant PRV YP2 strain can selectively replicate and lyse HER-2/neu-overexpressing human bladder, mouse bladder, and hamster oral cancer cells in vitro [13]. In the present study, our data show for the first time that different gene-deleted PRV vaccine strains Barthak61 and HB98 have anti-tumor effects on colon cancer.

To confirm the oncolytic potential of PRV, Bartha K61 and HB98 strain were used to infect colorectal cancer cell line HCT-8 and were found to cause CPEs, confirmed by titers of viral replication and cell viability. Both strains showed high cytotoxicity to HCT-8 cells with no significant difference from each other. Studies in vitro indicate that PRV can be used in further experiments in vivo.

In the in vivo experiment, mice were monitored during tumor progression, and the antitumor effects of intra-tumoral injection of Bartha K61 and HB98 strains were preliminarily evaluated. The results showed that both PRV Bartha K61 and HB98 strains could significantly inhibit tumor proliferation, and the oncolytic effect of the HB98 strain was more obvious. From the 12th day of treatment, there was a significant difference in the net body weight between the treatment group and the control group, the overall body weight of the treatment group was stable, while the rapid growth of tumors in the control group may affected the metabolism of protein, fat, and carbohydrates, thus inducing weight loss in mice. However, it is worth noting that the mice in the Bartha K61 treatment group began to itch and had skin ulcerate on the 14th day of treatment, and all died on the 16th day, while mice in the HB98 treatment group did not show any symptoms. These results suggest that the Bartha K61 vaccine strain may cause safety problems. From the point of view of virulence gene analysis, although the Bartha K61 vaccine strain carries deletions encoding gI, gE, US9, and US2 genes [23,29], in which gE and gI jointly affect virulence [30] and are necessary for the anterograde neural transport of virus particles [31], some studies have found that the PRV Bartha K61 strain can still cause death of mice at 9 days (220 h) after infecting both sides of mouse skin [25], and KM mice inoculated with 10^5^ TCID_50_ Bartha K61 developed clinical signs of disease that included pruritus and clawing and biting the injection site at 96 hpi. These mice died at 151 hpi and 177 hpi, respectively [32]. Our results are consistent with them in immunodeficient and immunocompetent mice. This shows that mice are susceptible to PRV, even if some virulence genes are deleted, Bartha K61 shows significant oncolytic effects and highly virulent to mice. PRV HB98, a triple gene deletion strain of TK^−^/gG^−^/gE^−^. PRV UL23 (TK) is not necessary for virus growth in most cultured cells, and UL23-negative PRV mutants are highly attenuated in mice, rabbits, and pigs [33,34]. BALB/c mice with a normal immune system were inoculated with 10^7^ TCID_50_ or 10^6^ TCID_50_ PRV HB98 strain, and the injections were repeated after 2 weeks, all the mice were still alive after 56 days [15,22]. Our study also showed that HB98 treatment did not cause morbidity and death in mice, and in the further in vivo safety test. It was again proven that the oncolytic effect of HB98 attenuated strain with low virulence was more obvious than the Bartha K61. Furthermore, the phenomenon we found in immunodeficient mice had similar results in immunocompetent mice. 

Although these two PRV strains are attenuated with different gene deletions, there was still concern that PRV may spread from the injection site to other tissues and cause viral infection. The results of PRV biodistribution in the nude mice transplantation model showed that PRV was mainly located at the injection site only, and a small amount of virus was detected in other organs only at 6 h after injection in the HB98 group; while in the Bartha K61 group, the virus was still detected in other tissues at 120 h after injection, indicating that Bartha K61 strain’s virulence is stronger, and prone to cause the infection in mice.

Pathological sections and immunohistochemistry were used to examine the morphological changes and to explore the potential mechanism of tumors after PRV treatment compared with the control group. Pathological sections showed that tumor cells in both HB98 and Bartha K61 groups formed large areas and a higher degree of necrosis, while the necrotic lesions in the control group may reflect the ischemic foci formed by the rapid growth of the tumor nodules and delayed neovascularization [24]. On the other hand, the larger focus of necrosis in the treatment group is caused by the virus, because it has an obvious locality. Although the virus was injected into the tumor at multiple points, it still cannot ensure that the virus was evenly distributed in the tumor, which means that we need to improve the injection method in the next step.

Apoptosis is an important cellular process that can be induced or inhibited by virus infection. Apoptosis plays an important role in the anti-tumor effect [35]. Previous studies have shown that apoptosis is involved in oncolysis induced by oncolytic virus [36,37,38]. There is evidence that HSV-1 can also induce apoptosis in human gastric cancer cells and HEp2 cells [26,27]. Thus, we explored the underlying mechanism of PRV-induced oncolysis by flow cytometry and IHC analysis, revealing that apoptosis may play an important role in PRV-induced cytotoxicity. Our data further demonstrated that PRV induced cell apoptosis by induction of the cleavage of caspase3. 

PRV has several advantages that make it a potential vector for cancer gene therapy. Firstly, PRV shares many similarities with HSV-1, and although neurotropic, it can infect a wide variety of host cells despite being neurotropic. Secondly, it can transduce non-dividing cells and dividing cells and can express transgene products with high efficiency. Finally, the large genome of PRV can accommodate several kilobases (kb) of foreign DNA, and the stable expression of foreign genes does not affect the stability of the virus itself. The positions of the TK, PK, gE, gI, and gG genes that are not essential for viral replication are possible insertion sites [39,40]. Studies have shown that the carcinoembryonic antigen (CEA) tumor promoter can functionally replace the PRV IE180 promoter, and the recombinant PRV-CEA virus can preferentially replicate in different cancer cell lines, and PRV-CEA can additionally induce apoptosis type [41]. This also provides ideas for the next step to transform PRV into an oncolytic carrier for further improving its targeting of tumors. It is worth thinking that PRV infects both human cancer cells and epithelial cells [11,42,43], which is the same as HSV. Therefore, how to enhance the targeting of cancer cells and the safety of the virus to cancer cells will be the focus of our future research.

Taken together, we demonstrate for the first time that the PRV can enter tumor cells in an HCT-8 cell-derived xenograft model and can effectively inhibit tumor growth. Our findings suggest that gene-deleted PRV strains could serve as the basis for the development of effective anti-tumor drugs, provides data on the anti-tumor activity of the oncolytic PRV, and justify the possibility of subsequent clinical trials of tumor therapy in non-primates and humans. 

## 5. Conclusions

In summary, the PRV gene-deleted vaccine strain inhibited the growth of both HCT-8 cells in vitro and BALB/c nu mice xenograft tumor models in vivo. The PRV HB98 strain showed a higher anti-tumor effect and was safer than the Bartha K61 strain in HCT-8 cells. The oncolytic PRV may induce apoptosis in HCT-8 cells and BALB/c nu mice xenograft tumor models. In this study, the results demonstrate that this oncolytic PRV HB98 strain has great potential as a new measure for anticancer therapy.

## Data Availability

Not applicable.
